# Inappropriate Timing of Swallow in the Respiratory Cycle Causes Breathing–Swallowing Discoordination

**DOI:** 10.3389/fphys.2017.00676

**Published:** 2017-09-22

**Authors:** Naomi Yagi, Yoshitaka Oku, Shinsuke Nagami, Yoshie Yamagata, Jun Kayashita, Akira Ishikawa, Kazuhisa Domen, Ryosuke Takahashi

**Affiliations:** ^1^Department of Swallowing Physiology, Hyogo College of Medicine Nishinomiya, Japan; ^2^Department of Neurology, Graduate School of Medicine, Kyoto University Kyoto, Japan; ^3^Clinical Research Center for Medical Equipment Development, Kyoto University Hospital Kyoto, Japan; ^4^Department of Physiology, Hyogo College of Medicine Nishinomiya, Japan; ^5^Department of Health Sciences, Prefectural University of Hiroshima Hiroshima, Japan; ^6^Graduate School of Health Sciences, Kobe University Kobe, Japan; ^7^Department of Physical Medicine & Rehabilitation, Hyogo College of Medicine Nishinomiya, Japan

**Keywords:** aspiration, coordination between breathing and swallowing, phase resetting, dysphagia, deglutition disorders

## Abstract

**Rationale:** Swallowing during inspiration and swallowing immediately followed by inspiration increase the chances of aspiration and may cause disease exacerbation. However, the mechanisms by which such breathing–swallowing discoordination occurs are not well-understood.

**Objectives**: We hypothesized that breathing–swallowing discoordination occurs when the timing of the swallow in the respiratory cycle is inappropriate. To test this hypothesis, we monitored respiration and swallowing activity in healthy subjects and in patients with dysphagia using a non-invasive swallowing monitoring system.

**Measurements and Main Results:** The parameters measured included the timing of swallow in the respiratory cycle, swallowing latency (interval between the onset of respiratory pause and the onset of swallow), pause duration (duration of respiratory pause for swallowing), and the breathing–swallowing coordination pattern. We classified swallows that closely follow inspiration (I) as I-SW, whereas those that precede I as SW-I pattern. Patients with dysphagia had prolonged swallowing latency and pause duration, and tended to have I-SW or SW-I patterns reflecting breathing–swallows discoordination.

**Conclusions:** We conclude that swallows at inappropriate timing in the respiratory cycle cause breathing–swallowing discoordination, and the prolongation of swallowing latency leads to delayed timing of the swallow, and results in an increase in the SW-I pattern in patients with dysphagia.

## Introduction

Breathing–swallowing coordination is one of the most important airway defense mechanisms (Nishino, 2012). Swallowing normally occurs during expiration, and the subsequent respiration reinitiates with expiration (Shaker et al., 1992; Martin et al., 1994; Martin-Harris et al., 2005). This expiration-swallow-expiration (E-SW-E) pattern prevents the pharyngeal contents from invading the lower airway. However, other swallowing patterns are observed even in healthy subjects, although the occasion is rare (Martin-Harris et al., 2005). Namely, swallowing occurs during inspiration (I-SW pattern) and inspiration occurs immediately after a swallow (SW-I pattern). The frequency of I-SW and SW-I patterns increases with age, in patients with stroke (Leslie et al., 2002), head-neck cancer after treatment (Gillespie et al., 2005), Parkinson's disease (Gross et al., 2008), and chronic obstructive pulmonary disease (COPD) (Gross et al., 2009). An increase in the discoordination between breathing and swallowing may predispose patients to aspiration pneumonia and exacerbation of COPD. Indeed, an increase in I-SW pattern is associated with a risk of aspiration in Parkinson's disease (Troche et al., 2011). Recently, we reported that frequent I-SW and/or SW-I patterns (high I-SW/SW-I rate) exacerbated COPD (Nagami et al., 2017). Therefore, the identification of such subjects with a high I-SW/SW-I rate and treating them to reduce the I-SW/SW-I rate may prevent exacerbations of these diseases.

The present study was aimed to gain insight into the mechanisms underlying I-SW and/or SW-I patterns, and to determine a possible means to reduce the I-SW/SW-I rate via various types of interventions. We hypothesized that breathing–swallowing discoordination occurs when the timing of the swallow in the respiratory cycle is inappropriate. The concept in which swallowing is considered as a perturbation to the respiratory cycle was originally proposed by Paydarfar et al. (1995). Although, they reported that the period of expiration is the shortest when swallows are initiated near the expiratory-to-inspiratory transition, they did not describe whether the swallowing is followed by inspiration or expiration. In addition, it is not clear whether the increase in the I-SW/SW-I rate in patients with dysphagia is accompanied by shifts in the timing of swallowing in the respiratory cycle, and most importantly, why such shifts in timing occur. To clarify these issues, we monitored the parameters associated with breathing–swallowing coordination in healthy subjects and in patients with dysphagia, using a non-invasive swallowing measurement system (Yagi et al., 2016).

## Materials and methods

The study protocol has been approved by local ethical committees of Hyogo College of Medicine (No. 1580) and Kyoto University (No. C819). All subjects gave written informed consent in accordance with the Declaration of Helsinki. We recruited 250 volunteer subjects from Daito City Cohort, who regularly participate in community-operated exercise program for care prevention, and 43 volunteer subjects who visited a health promotion festival at Ashiya Municipal Hospital. Subjects who have a history of aspiration pneumonia, manifest clinically evident cerebrovascular or respiratory disease, or have medication with dopaminergic drugs were excluded. We also recruited 30 patients with dysphagia (stable subacute–chronic phase of illness) who were hospitalized for swallowing rehabilitation. Characteristics of these patients are shown in Table 1. Underlying diseases included stroke, intracranial hemorrhage, tuberculous spondylitis, pneumonia, myocardial infarction, and unknown etiology (possible frail condition or sarcopenia). Dysphagia in stroke patients was classified as upper motor neuron lesions in all cases, however, a detailed evaluation with regard to the location of stroke (e.g., whether it included swallow-related cortical areas) was not undertaken, because stroke location is not typically related to the risk of aspiration (Steinhagen et al., 2009; Daniels et al., 2017).

**Table 1:** Characteristics of 30 subjects with dysphagia.

**No.**	**Underlying diseases**	**Gender**	**Age**	**BMI**
1	Aortic dissection, post-operative state, suspect of left recurrent nerve palsy	F	56	20.8
2	Brainstem infarction (left ventral pons)	M	77	22.0
3	Cerebral hemorrhage (right putamen)	F	79	18.6
4	Cerebral hemorrhage (right thalamus)	M	81	19.0
5	Cerebral infarction	F	76	28.5
6	Cerebral infarction (left temporal lobe)	M	63	23.9
7	Cervical spondylotic myelopathy	F	71	24.9
8	Encephalitis	M	55	25.3
9	Myocardial infarction	M	77	24.0
10	Old cerebral infarction	M	81	21.8
11	Organizing pneumonia	M	78	22.3
12	Right femoral trochanter fracture	F	70	17.4
13	Subcortical infarction	F	67	24.2
14	Tracheostomized state	M	58	18.8
15	Tuberculous spondylitis	M	72	19.9
16	Unknown etiology	F	84	29.9
17	Unknown etiology	F	82	22.3
18	Right carotid endarterectomy, post-operative state	M	89	22.3
19	Parkinson's disease	M	74	18.7
20	Cerebral infarction (left corona radiata, right frontal lobe), dementia with Lewy body, diabetes mellitus	F	87	19.1
21	Brain contusion (left frontal lobe)	F	76	16.0
22	Multiple brain infarction	M	55	18.5
23	Multiple brain infarction	F	69	18.1
24	Left subarachnoid hemorrhage	F	70	20.8
25	Cardiogenic brain embolism	M	65	19.4
26	Cerebral hemorrhage (right thalamus)	M	62	16.9
27	Cerebral infarction (right corona radiata)	M	73	19.0
28	Brain tumor	F	88	16.5
29	Cerebral hemorrhage (left putamen)	F	85	21.5
30	Cerebral hemorrhage	F	80	17.9
		M:15 F:15	73.3 ± 9.9 (range: 55–89)	20.9 ± 3.4 (range: 16.0–29.9)

### Evaluation of swallowing function

Dysphagia was diagnosed if any of the following four criteria was met:

1. Food intake level scale (FILS) (Kunieda et al., 2013) less than Level 10. This scale categorizes the severity of dysphagia primarily based on methods of feeding. Subjects who receive nutrition via non-oral pathways are categorized as Levels 1–3, subjects who receive nutrition by both oral dysphagia diet and alternative nutrition are categorized as Levels 4–6, subjects who receive nutrition by various degrees of dysphagia diet are categorized as Levels 7–9, and normative subjects are categorized as Level 10.
2. Repetitive Saliva-Swallowing Test (RSST) (Hori et al., 2016), a count of swallows over a 30-s period, <3. In this test, subjects are instructed to swallow saliva as many times as possible within 30 s. An RSST count of less than three times in 30 s suggests suspected dysphagia, and the sensitivity and specificity of RSST to predict aspiration in videofluorographic examination are reported to be 0.98 and 0.66, respectively (Oguchi et al., 2000).
3. Water swallowing test (WST) >1. In this test, subjects are instructed to drink 30 ml of water from a cup, and the condition of swallowing is scored. Score 1 indicates a normative swallowing function (ability to swallow 30 ml water at once in 5 s), whereas Score 5 indicates the worst swallowing function (frequent cough or inability to swallow) (Zhang et al., 2016; Table 2).
4. Modified water swallowing test (MWST) score <5. In this test, subjects are instructed to swallow 3 ml of water, which is injected into the subject's oral cavity with a syringe, and the condition of swallowing is scored. If the subject is unable to swallow, or experienced dyspnea, coughing, or wet-hoarse dysphonia after swallowing, a score of 1–3 is recorded and the test is discontinued. If the subject is able to swallow the water, the subject is asked to perform two dry (saliva) swallows. If the patient is able to complete the water and both dry swallows, a score of 5 is recorded, otherwise, a score of 4 is recorded (Tohara et al., 2003; Murakami et al., 2015; Table 3). The sensitivity and specificity of MWST to differentiate between aspirators and non-aspirators with a cutoff level of 3 are 70 and 88%, respectively (Tohara et al., 2003).

**Table 2:** Thirty milliliter Water swallow test (WST).

**Score**	**Performance**
1	Swallow water at once in 5 s without cough
2	Swallow more than twice, without cough
3	Swallow water at once, but with cough
4	Swallow more than twice, but with cough
5	Cough frequently, or inability to swallow water

**Table 3:** Three milliliter Modified water swallow test (MWST).

**Score**	**Performance**
1	Inability to swallow with choking and/or breathing changes
2	Swallow occurred, but with breathing changes
3	Swallow occurred, but with choking and/or wet hoarseness
4	Swallow successfully
5	Swallow successfully with ability of additional dry swallowing twice in 30 s

FILS was recorded for all patients, and all subjects scored <10 (7.3 ± 2.8). Since the severity of dysphagia in these patients was mild, each subject additionally underwent at least one of three swallowing screening tests to confirm the functional impairment (RSST: 2.2 ± 1.3, *n* = 28; MWST: 4.4 ± 0.5, *n* = 14; WST: 1.9 ± 0.3, *n* = 10).

For those who were recruited from the Daito City Cohort and at the health promotion festival, the Japanese version of the 10-item eating assessment tool (EAT-10) was performed to facilitate clear differentiation of healthy subjects within the elderly population. EAT-10 score of 3 or higher was considered as possible dysphagia (Belafsky et al., 2008). According to this criterion, 269 subjects belonged to the healthy group, and 24 subjects belonged to the possible dysphagic group. The mean ages of the healthy, possible dysphagic group and of the dysphagic group were not statistically different (healthy: 75.0 ± 6.1 years old, range 56–92 years old, 21 males and 248 females, possible dysphagia: 75.9 ± 6.6 years old, range 66–96 years old, 3 males and 21 females, dysphagia: 73.3 ± 9.9 years old, range 55–89 years old, 15 males and 15 females, *p* = 0.324).

### Monitoring of swallowing

To evaluate breathing–swallowing coordination, we developed a unique swallow monitoring device with minimal instrumentation, which enabled even ambulant monitoring of the swallowing function (Yagi et al., 2016). The device consists of a nasal cannula-type flow sensor connected to a differential pressure transducer, a film-type piezoelectric sensor attached on the surface of the thyroid cartilage, and a logging system that stores signals in a microSD card. The piezoelectric sensor has a wide dynamic range (0–4 kHz) so that both the laryngeal motion and sound are captured. Swallowing periods are extracted semi-automatically with an algorithm using the respiratory flow, the swallowing sound, and the laryngeal motion.

**Figure 1:**
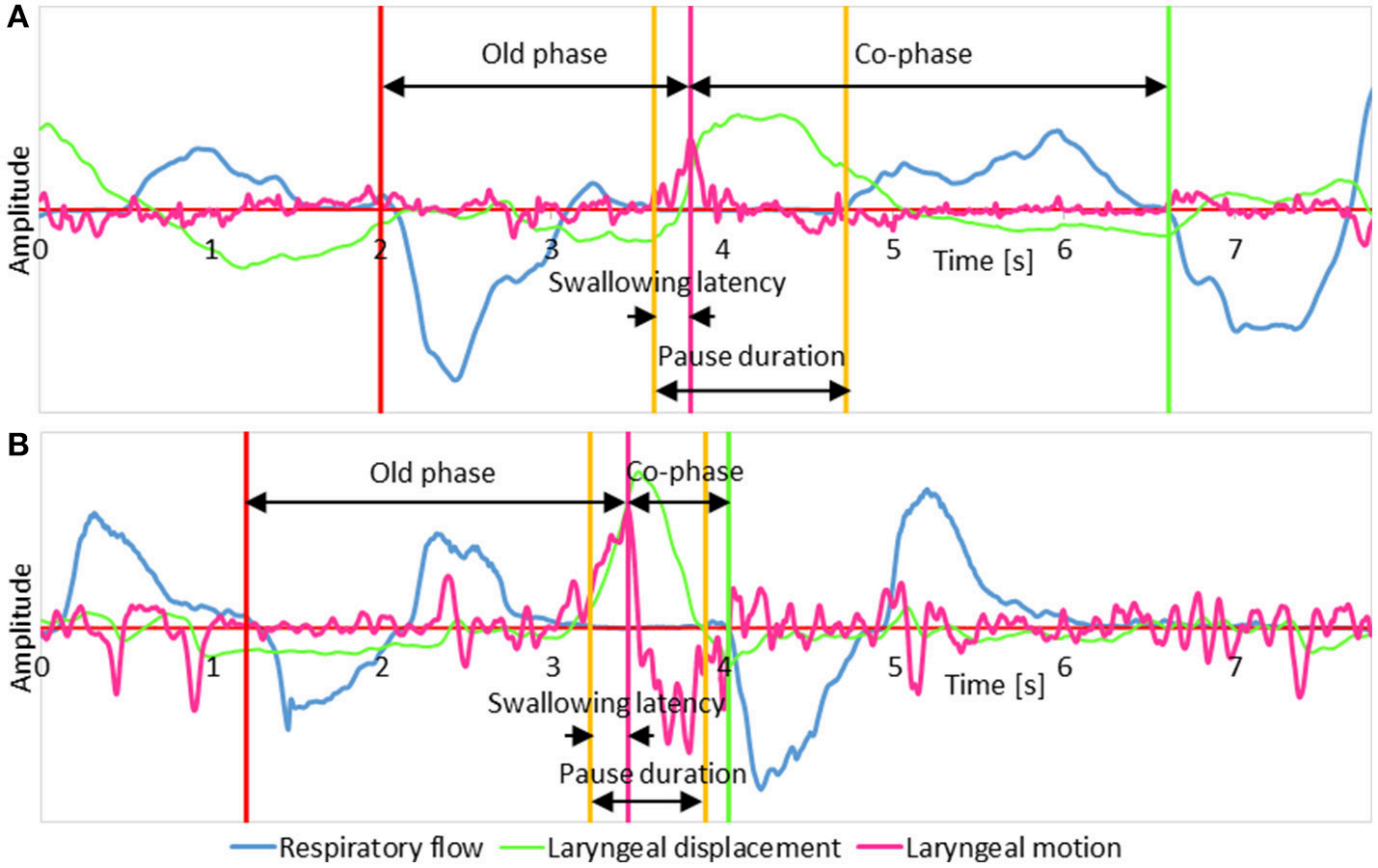
The schema illustrates parameters measured on signal traces. **(A)** A representative case of a swallow with the expiration-swallow-expiration pattern; **(B)** A representative case of a swallow with the expiration-swallow-inspiration pattern. In both traces, respiratory flow, laryngeal motion, and laryngeal displacement signals are shown in blue, magenta, and green, respectively. A positive shift in the respiratory flow signal represents expiration, and a negative shift represents inspiration. The laryngeal displacement signal was derived by integrating the laryngeal motion signal.

We analyzed the following parameters associated with breathing–swallowing coordination, as shown in Figure 1, using custom-made programs, written in MATLAB R2014b (Mathworks, Natick, MA, USA). A total of 2,648 swallows were analyzed.

1. Old phase
  The timing of swallow in the respiratory cycle, which is expressed as the time from the preceding inspiration normalized by the mean length of the respiratory cycle being 1 (Paydarfar et al., 1995).
2. Co-phase
  The time from the swallowing onset to the immediately following inspiration normalized by the mean length of the respiratory cycle being 1 (Paydarfar et al., 1995).
3. Pause duration
  The duration of respiratory pause associated with swallowing. The respiratory pause for voluntary swallow is highly variable (Palmer and Hiiemae, 2003; Matsuo et al., 2008), and thus we avoid using the term “deglutition apnea,” which is regulated by interactions between the central pattern generators of breathing and swallowing in the brainstem (Oku et al., 1994).
4. Swallowing latency
  The time from the onset of respiratory pause to the onset of the swallowing reflex, defined as the time point when the speed of the laryngeal elevation reaches the maximum (Yagi et al., 2016).
5. The time from the onset of swallow to the next inspiration.
6. Breathing–swallowing coordination pattern
  We categorized the breathing–swallowing pattern using two types of parameters: (1) B-SW type, a parameter describing the combination of swallow and the preceding respiratory phase, either E-SW (expiration-swallow) or I-SW (inspiration-swallow), and (2) SW-B type, a parameter describing the combination of swallow and the following respiratory phase, either SW-E (swallow-expiration) or SW-I (swallow-inspiration).

### Test foods

We used soft jelly and water for test foods. The properties of the soft jelly, i.e., hardness, adhesiveness, and cohesiveness are strictly controlled to meet the criteria of Level 0 dysphagia diet according to the Japanese Society of Dysphagia Rehabilitation (JSDR) specification described in IDDSI (International Dysphagia Diet Standardization Initiative) report (Cichero et al., 2016). Subjects were in an upright position sitting on a chair, and swallowed voluntarily about 3 g of Level 0 test food from a teaspoon and 3 ml of water from a 5-ml syringe, two to five times for each. Subjects were instructed to swallow the Level 0 jelly without chewing.

### Statistical analysis

The correlations between parameters were evaluated using Pearson's product-moment correlation analysis. Comparisons of the parameters between different breathing–swallowing coordination types were performed using single-measurement, simple-factorial analysis of variance (ANOVA), followed by *post hoc* analyses with Tukey-Kramer test. Differences in the frequency of breathing–swallowing coordination patterns between three different timings of swallow in the respiratory cycle, and differences in timings of swallow in the respiratory cycle between healthy, possible dysphagic, and dysphagic groups were compared using the chi-squared test followed by Haberman's residual analysis. All data are presented as mean ± standard deviation. *P*-values were two-sided, and *P* < 0.05 was considered as statistically significant.

## Results

### Correlations between breathing–swallowing coordination parameters

**Figure 2:**
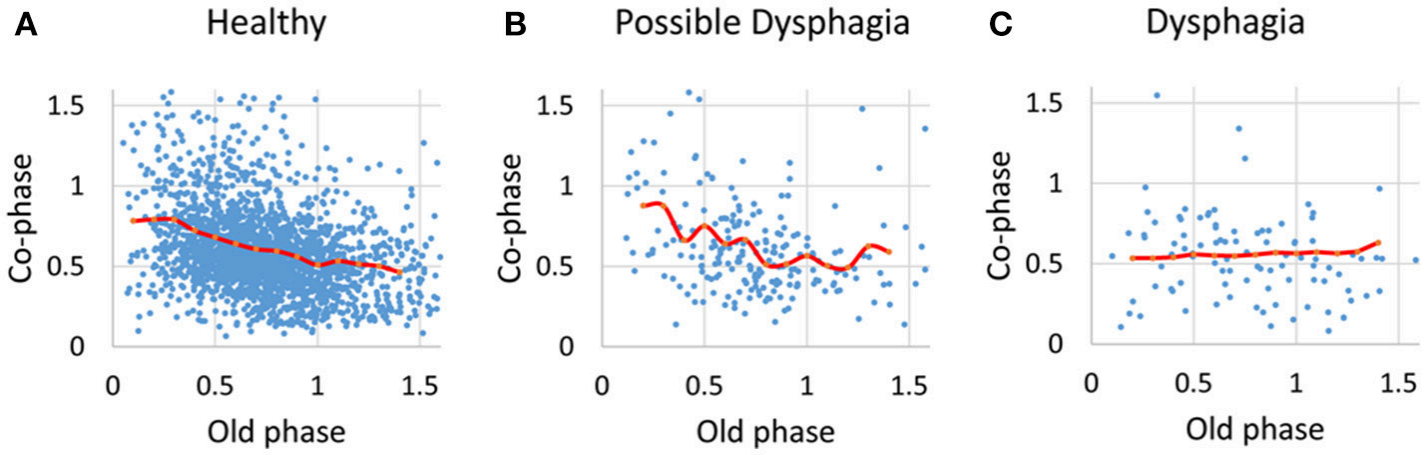
The relationships between old phase and co-phase are plotted for healthy subjects **(A)**, possible dysphagic subjects **(B)**, and dysphagic subjects **(C)**. Red lines indicate the phase-response curves for each subject group calculated by averaging the co-phase within a bin (bin width = 0.1).

**Figure 3:**
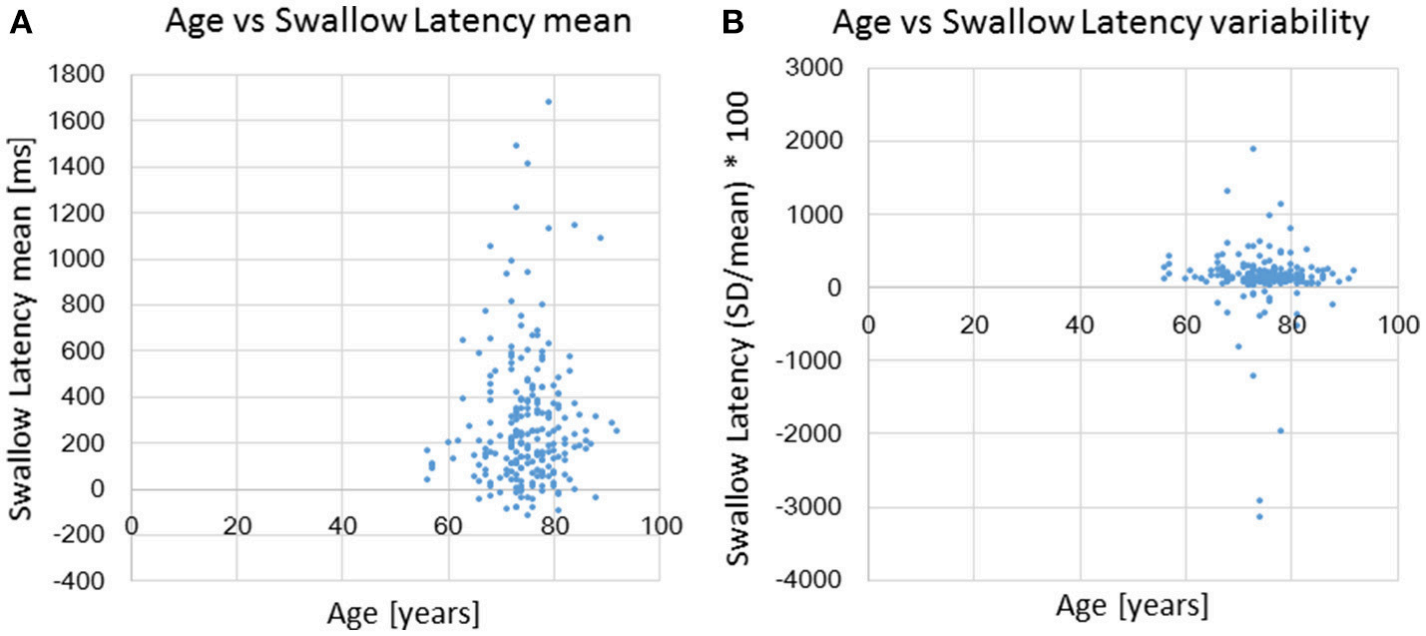
The relationships between age and swallowing latency **(A)** and age and coefficient of variation [(*SD*/mean) ^*^ 100] of swallowing latency **(B)**.

For analyses in this subsection, we utilized all data from the healthy, possible dysphagic, and dysphagic groups. We found a strong correlation between swallowing latency and pause duration (*r* = 0.867, *p* < 0.0001; Table 4), and weak correlations between old phase and swallowing latency (*r* = 0.316, *p* < 0.0001), between old phase and pause duration (*r* = 0.216, *p* < 0.0001), and between old phase and I-SW frequency (*r* = −0.259, *p* < 0.0001). The swallowing latency was variable, which resulted in scattered phase resetting characteristics shown as a co-phase plot (Figure 2). To gain insight into the variability of swallowing latency and pause duration, we quantified the variability of swallowing latency and pause duration (deglutition apnea) for those who had more than eight swallow records. Neither swallowing latency nor variability [coefficient of variation; (*SD*/mean) ^*^ 100] of swallowing latency was correlated with age (Figure 3). Further, neither pause duration nor variability of pause duration was correlated with age.

**Table 4:** Correlations between parameters associated with breathing–swallowing coordination.

	**Old phase**	**Co-phase**	**Swallow latency**	**Pause duration**	**I-SW frequency**	**SW-I frequency**
Old phase	1.000	0.041	0.316##	0.216##	−0.259##	0.202#
Co-phase	0.041	1.000	−0.033	0.039	0.173^**^	−0.388##
Swallow latency	0.316##	−0.033	1.000	0.867##	0.189#	0.029
Pause duration	0.216##	0.039	0.867##	1.000	0.203#	0.056
I-SW frequency	−0.259##	0.173^**^	0.189#	0.203#	1.000	−0.026
SW-I frequency	0.202#	−0.388##	0.029	0.056	−0.026	1.000

** *p < 0.01*,

# *p < 0.001*,

## *p < 0.0001*.

Swallows with E-SW-I pattern had a significantly larger old phase, smaller co-phase, and shorter time to the following inspiration as compared to those with E-SW-E pattern, whereas swallows with I-SW-E pattern had a significantly smaller old phase, larger co-phase, and longer time to the following inspiration (Table 5). To further elucidate the relationship between old phase and breathing–swallowing discoordination, we divided the old phase into three groups, early (old phase <0.4), intermediate (old phase between 0.4 and 1.0), and late (old phase >1.0). Swallows with an early old phase had a greater chance of I-SW pattern as compared to those with intermediate and late old phases, whereas swallows with a late old phase had a greater chance of SW-I pattern as compared to those with early and intermediate old phases (Table 6). This relationship between old phase and breathing–swallowing discoordination was common in both healthy subjects and patients with dysphagia, suggesting that the characteristic does not depend on a specific etiology of dysphagia.

**Table 5:** Comparisons of parameter values between different breathing-swallowing coordination patterns.

	**E-SW-E**	**E-SW-I**	**I-SW-E**	**I-SW-I**
*N*	2,197	199	242	10
Old phase	0.790 ± 0.377	1.019 ± 0.576##	0.483 ± 0.308##	0.229 ± 0.133##
Co-phase	0.617 ± 0.298	0.232 ± 0.080##	0.836 ± 0.320##	0.251 ± 0.083#
Swallow latency (ms)	244 ± 466	281 ± 693	624 ± 787##	162 ± 288
Pause duration (s)	0.911 ± 0.578	0.992 ± 0.749	1.252 ± 0.873##	0.941 ± 0.407
Time to the next inspiration (s)	2.385 ± 1.672	0.777 ± 0.242##	3.268 ± 1.546##	0.844 ± 0.267^*^

Tukey-Kramer test P-value to E-SW-E (

* *p < 0.05*,

# *p < 0.001*,

## *p < 0.0001)*.

**Table 6:** Frequency distributions of different breathing-swallowing coordination patterns in three timings of swallow.

**Old phase type**	**E-SW-E**	**E-SW-I**	**I-SW-E**	**I-SW-I**
Early	53.5 (154)##	2.1 (6)^**^	41.3 (119)##	3.1 (9)##
Intermediate	82.0 (1602)	14.5 (115)	3.5 (104)	0.0 (1)
Late	87.9 (441)#	6.3 (78)##	5.7 (19)^*^	0.1 (0)

*Frequencies are indicated as percent. Values in parentheses indicate numbers of swallow*.

Haberman test P-value to Intermediate (

* *p < 0.05*,

** *p < 0.01*,

# *p < 0.001*,

## *p < 0.0001)*.

### Comparison of parameters between healthy subjects, patients with dysphagia, and possible dysphagic subjects

**Figure 4:**
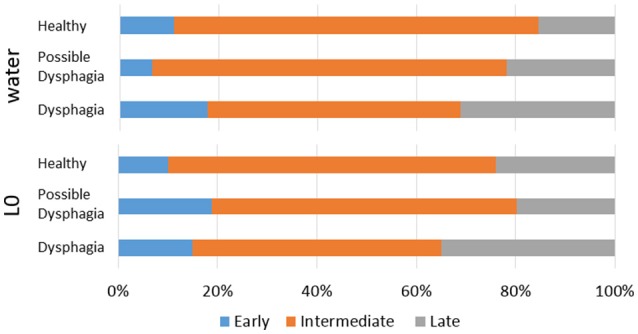
Distributions of timings of swallow in the respiratory cycle in healthy, possible dysphagic, and dysphagic subjects. Upper panel: water swallows; Lower panel: Level 0 dysphagia diet (L0) swallows.

Parameters associated with breathing–swallowing coordination in healthy, dysphagic, and possible dysphagic groups are listed in Table 7. When we put together all swallows from Level 0 and water swallows, the old phase, pause duration, and swallowing latency were significantly larger in dysphagic group, however in possible dysphagic group, only pause duration was significantly larger as compared to healthy group. Further, the frequencies of E-SW-I and I-SW-I patterns were significantly higher in dysphagic group, but not in possible dysphagic group, as compared to healthy group. Timings of swallow in patients with dysphagia were shifted to either early or late timings (Figure 4, Table 8). The difference in the timings of swallow and the I-SW frequency between healthy and dysphagic groups was more marked in water swallows.

**Table 7:** Comparisons of parameter values and frequencies of breathing-swallowing coordination patterns between healthy, possible dysphagic, and dysphagic groups.

		**Healthy**	**Possible dysphagia**	**Dysphagia**
*N*		2,327	216	105
Old phase		0.770 ± 0.376	0.776 ± 0.381	0.946 ± 0.837##
Pause duration (s)		0.931 ± 0.597	1.059 ± 0.803^*^	1.099 ± 0.886^*^
Swallow latency (ms)		271 ± 506	332 ± 674	413 ± 750^*^
Swallow type (%) (*N*)	E-SW-E	83.8 (1950)	79.2 (171)	72.4 (76)^**^
	E-SW-I	7.0 (163)	9.3 (20)	15.2 (16)^**^
	I-SW-E	8.9 (208)	11.6 (25)	8.6 (9)
	I-SW-I	0.3 (6)	0.0 (0)	3.8 (4)##

* *p < 0.05*,

** *p < 0.01*,

## *p < 0.0001*.

*Tukey-Kramer test P-value to Healthy in Old phase, Pause duration, and Swallow latency*.

*Haberman test P-value to Healthy in Swallow type*.

Table 8Comparisons of timings of swallow and frequencies of breathing-swallowing coordination for different types of test foods.

**Table:** 

**Test type**		**Old phase type**	**Early**	**Intermediate**	**Late**
L0	frequency (%) (*N*)	Healthy	10.1 (119)	66.0 (778)	23.9 (281)
		Possible dysphasia	18.9 (21)^**^	61.3 (68)	19.8 (22)
		Dysphagia	15.0 (9)	50.0 (30)^*^	35.0 (21)^*^
Water	frequency (%) (*N*)	Healthy	10.8 (124)	73.8 (848)	15.4 (177)
		Possible dysphasia	6.7 (7)	71.4 (75)	21.9 (23)
		Dysphagia	17.8 (8)	51.1 (23)#	31.1 (14) ^**^

**Table:** 

**Test Type**		**Healthy**	**Possible Dysphasia**	**Dysphagia**
L0	*N*	266	24	29
	I-SW frequency (%)	10.5 ± 18.2	16.0 ± 17.5	5.2 ± 20.5
	SW-I frequency (%)	6.3 ± 17.8	8.1 ± 15.0	12.9 ± 24.7
Water	*N*	265	24	20
	I-SW frequency (%)	7.7 ± 16.4	6.3 ± 12.4	19.8 ± 28.8^**^
	SW-I frequency (%)	9.4 ± 19.3	11.0 ± 21.7	18.5 ± 29.1

Haberman test P-value to Healthy (

* *p < 0.05*,

** *p < 0.01*,

# *p < 0.001); L0: Level 0 dysphagia diet (Matsuo et al., 2008)*.

### Correlation between breathing–swallowing discoordination and severity of dysphagia

We evaluated correlations between parameters associated with breathing–swallowing coordination and those associated with severity of dysphagia to determine whether breathing–swallowing discoordination is deteriorated in proportion to the severity of dysphagia (Table 9). A significant negative correlation was found between MWST and SW-I frequency. MWST of subjects in the present study was either 4 or 5 points, thus the result implies that patients who could swallow twice additionally within 30 s after 3 ml water swallow (5 points) had fewer SW-I patterned swallows. Significant negative correlations were also found between WST and I-SW or SW-I frequency. WST of subjects in the present study was either 1 or 2 points, thus the result implies that patients who could swallow water at once in 5 s had, ironically, a higher chance of I-SW or SW-I swallow patterns as compared to those who needed to swallow more than twice. FILS, RSST, and EAT-10 were not correlated with I-SW frequency or SW-I frequency, indicating that these dysphagia screening tools could not detect breathing–swallowing discoordination.

**Table 9:** Correlations between severity of dysphagia and parameter values associated with breathing-swallowing coordination.

	**EAT-10**	**FILS**	**RSST**	**MWST**	**WST**
N	298	30	28	14	10
Old phase	0.138^*^	−0.065	−0.190	−0.333	0.432
Co-phase	−0.038	−0.178	−0.248	0.671^**^	0.343
Swallow latency	0.032	−0.358	−0.327	−0.325	0.072
Pause duration	0.046	−0.286	−0.325	−0.119	−0.023
Time to the next inspiration	−0.025	−0.300	−0.447^*^	0.515	0.434
I-SW frequency	−0.027	0.163	0.081	−0.198	−0.890#
SW-I frequency	0.105	0.073	0.195	−0.628^*^	−0.646^*^

(* *p < 0.05*,

** *p < 0.01*,

# *p < 0.001)*.

### Logistic analysis to discriminate healthy subjects and patients with dysphagia

We performed univariate and multivariate logistic regression analyses in an attempt to discriminate healthy subjects and patients with dysphagia using parameters associated with breathing–swallowing coordination. For the logistic analysis, we introduced a new variable, the frequency of “old phase out of range,” which was defined as the old phase either within the range of early old phase (<0.4) or late old phase (>1.0). We calculated the area under the curve (AUC) of the receiver operating characteristic (ROC) curve to evaluate the model performance for different combinations of parameters. The model parameters were selected from those having a small *p*-value in univariate regression analysis (Table 10). We found that the combination of SW-I frequency, the frequency of old phase out of range, and pause duration showed the best performance (AUC = 0.730) in multivariate logistic regression analyses.

**Table 10:** Univariate and multivariate regression analyses to differentiate between healthy and dysphagic subjects.

	**Univariate logistic regression *P*-value**	**Multivariate logistic regression *P*-value**	**AUC**
SW-I frequency	0.052	0.024	0.730
Old phase out of range	0.023	0.008	
Pause duration	0.002	0.002	

## Discussion

The new findings of the present study are the following: (1) swallows with either early or late old phase are likely to be an uncoordinated breathing–swallowing pattern in both healthy and patient groups, (2) the old phase of patients with dysphagia is delayed as compared to that of healthy subjects, and (3) respiratory pause duration, highly coupled with swallowing latency, is prolonged in patients with dysphagia. These results suggest that the breathing–swallowing discoordination tends to occur when the timing of swallow in the respiratory cycle is either advanced or delayed, and the prolongation of swallowing latency would cause the delay in timing of swallow in the respiratory cycle, and consequently result in an increase in SW-I pattern in patients with dysphagia. Therefore, interventions to shorten the swallowing latency may be a target of treatment.

### Relationship between breathing–swallowing coordination and timing of swallows relative to respiratory phases

When an intrinsic oscillator is perturbed by external stimuli, the timing at which a specific phase initiates is shifted (either advanced or delayed). The amount of phase shift depends on the timing and strength of the stimulus; the relationship between timing and amount of shift is termed phase resetting characteristic, which characterizes the oscillator (Glass and Mackey, 1988; Oku and Dick, 1992). Swallows are one of natural perturbations of the respiratory cycle. Paydarfar et al. (1995) investigated the relationship between timing of swallows and phase shift of the respiratory timing, and found that swallows strongly reset the respiratory rhythm (type-0 resetting, Glass and Mackey, 1988), and swallows at the expiratory-to-inspiratory phase transition have the smallest co-phase (i.e., the shortest time to the next inspiration), and swallows at early expiration have the longest co-phase (i.e., the longest time to the next inspiration). Although they did not discuss the frequency of SW-I pattern, the shortest time to the next inspiration for swallows at the expiratory-to-inspiratory phase transition implies an increase in chance of SW-I pattern, which is consistent with the results of the present study.

The major difference between phase resetting by involuntary swallowing and that by voluntary swallowing in the present study is the variability of swallowing latency in voluntary swallowing. It has been reported that the onset of respiratory pause for swallowing is approximately coincident with the bolus propulsion of liquid into the pharynx (Martin-Harris et al., 2005), however, the pause duration is highly variable when eating solid foods (Palmer and Hiiemae, 2003; Matsuo et al., 2008). Even with water swallows, we observed a considerable variability in swallowing latency. The pause in breathing often began substantially before the swallow, and the old phase sometimes exceeded the whole respiratory cycle length. We think that this is because voluntary swallowing is controlled by the cortex, while the occurrence of involuntary swallow in the respiratory cycle is regulated by the interaction between the central pattern generators of breathing and swallowing within the brainstem. We assume that the variability of cortically-controlled swallowing latency causes the scattering of the co-phase plot and breathing–swallowing discoordination.

### Neuronal mechanisms of respiratory phase resetting

By using transgenic mice in which Channelrhdopsin-2 (ChR2) or Archaerhodopsin (Arch) is specifically expressed in glycinergic neurons, it has been recently shown that activation of glycinergic neurons in the pre-Bötzinger complex (preBötC) leads to interruption of respiratory activity, whereas silencing of glycinergic neurons in the preBötC induces inspiration (Sherman et al., 2015). Simulation using a neural mass model consisting of five types of respiratory neurons in the Bötzinger Complex (BötC), the pre-BötC, and the rostral ventrolateral respiratory group (rVRG) could reproduce the experimental results (Oku and Hulsmann, 2017). On the other hand, the nucleus tractus solitarii (NTS) is the primary relay nucleus which receives afferent signals from pharyngeal mechanoreceptors and chemoreceptors primarily via the superior laryngeal nerve (SLN) (Jean, 2001; Miller, 2008). What we still do not know is the relay pathway from the solitary nucleus to respiratory neurons in the BötC and preBötC, and the types of glycinergic respiratory neurons that are activated or inhibited by the afferent signals. Neurons orthodromically activated by electrical stimulation of the SLN, which exhibited burst firing at the onset of swallowing, project to NTS, the nucleus ambiguus, the hypoglossal nucleus, the medullary reticular formation, and the dorsal motor nucleus of the vagus (Ezure et al., 1993; Sugiyama et al., 2011). These neurons may be involved in the phase resetting associated with swallowing.

### Relationship between pause duration and swallowing latency

We found a strong correlation between the duration of respiratory pause for swallowing and the swallowing latency. This relationship holds true regardless of the type of food, and regardless of whether the subjects are in healthy, dysphagic, or possible dysphagic groups. We do not know the neural mechanisms of this tight coupling, or mechanisms by which the duration of respiratory pause is regulated. One of the reasons for the strong correlation might be the fact that, as the respiratory pause lengthens, the swallowing latency occupies the majority of the respiratory pause.

As we discussed in the previous subsection, a swallow perturbs the respiratory cycle, and shifts the timing of the following inspiration depending on the timing in the respiratory cycle that the swallow occurred, however, this phase resetting theory does not consider factors associated with swallowing latency and pause duration. Paydarfar et al. (1995) reported that the duration of respiratory pause does not depend on the timing of swallow within the respiratory cycle. However, we found that both swallowing latency and pause duration are weakly correlated with the old phase, the timing of swallow in the respiratory cycle. Our analysis indicates that the swallowing latency and pause duration lengthen as the old phase becomes greater, however, interestingly, swallows with I-SW-E pattern, i.e., with early old phase, also had a longer swallowing latency and longer pause duration. This issue is further discussed below.

### Comparison between healthy subjects and patients with dysphagia

We found that the timing of swallows is delayed in patients. Considering that a swallow with a delayed timing tends to be SW-I pattern, we suggest that the increase in SW-I pattern in patients is the consequence of the delayed timing of swallows in the respiratory cycle.

We found that the duration of respiratory pause for swallowing is prolonged in patients with dysphagia. This is consistent with the study of Wang et al. (2015). We also found that the swallowing latency is prolonged in patients, and the pause duration was closely correlated with swallowing latency both in healthy subjects and in patients with dysphagia. It is known that the onset of swallowing reflex relative to a bolus invasion into the pyriform recess is delayed in patients with dysphagia (Miyaji et al., 2012). In addition, the time for food to be propelled into the pharynx may also be delayed in patients with dysphagia. We therefore suggest that the prolongation of the pause duration is a consequence of the delayed onset of swallowing reflex (relative to the respiratory phase) in patients with dysphagia.

Interestingly, we found that the swallowing latency as well as the pause duration are lengthened in swallows with I-SW-E pattern as compared to E-SW-E swallows. Therefore, I-SW-E swallows may be an adaptive behavior to compensate for the delayed onset of swallowing reflex. In contrast, I-SW-I pattern seen in patients with dysphagia would be unsafe swallows, since the time to the next inspiration is much shorter than that in swallows of E-SW-E pattern.

### Breathing–swallowing discoordination and severity of dysphagia

Currently, EAT-10, RSST, and WST are widely used for screening dysphagia. The FILS is generally used to describe the severity of dysphagia in Japan (Kunieda et al., 2013). It is noteworthy in that none of these tests are correlated with either I-SW frequency or SW-I frequency. This might be because patients we recruited had relatively mild dysphagia. Further studies are needed to elucidate whether breathing–swallowing discoordination correlates with the severity of dysphagia. Nevertheless, it should be noted that for patients with mild dysphagia, breathing–swallowing discoordination cannot be predicted by screening tools such as EAT-10, RSST, or WST.

### Differentiation between healthy subjects and patients with dysphagia

We found that the combination of SW-I frequency, the frequency of old phase out of range, and pause duration was the best logit model to differentiate between healthy and dysphagic subjects. However, AUC of this model was only 0.730, suggesting that there is considerable overlap between healthy and dysphagic groups. Indeed there were a number of healthy subjects who showed a high frequency of I-SW/SW-I patterns. We do not know whether these subjects will manifest symptoms of dysphagia as they become older. Discoordination between breathing and swallowing may be a personal trait, which is modified by age and disease (e.g., stroke, Parkinson's disease, and COPD), and could be corrected by rehabilitation (Martin-Harris et al., 2015).

### Limitations and future perspectives

Although we focused on the discoordination between breathing and swallowing, multiple factors affect the vulnerability of aspiration during swallowing. These include anatomical abnormalities and physiological impairments associated with the triggering of the pharyngeal stage, pharyngeal motor response, and the esophageal stage of swallowing (Lundy et al., 1999). Troche et al. (2010) showed that increasing expiratory muscle strength attenuates aspiration without changing the timing of swallow, presumably due to improved hyolaryngeal complex movement. Therefore, comprehensive measures should be taken to ameliorate the vulnerability for aspiration.

Coughing is another, and probably the most important airway defensive mechanism. The coordination of breathing, swallowing, and coughing has been recently studied by a group of the University of Florida (Pitts et al., 2012, 2013; Bolser et al., 2013). Although we did not record coughs, these studies are a natural extension exploring the mechanisms of airway protective reflexes, and should be further extended to human studies.

We tested only 30 patients with dysphagia. In addition, these patients had diverse etiologies, and were poorly characterized, which might have obscured the unique manifestations of swallowing-breathing coordination in patients with specific diseases or etiologies. Therefore, a future direction of study would be to test a sufficient number of patients with a uniform etiological background.

## Conclusion

In summary, breathing–swallowing discoordination occurs when the timing of swallow in the respiratory cycle is inappropriate. In patients with dysphagia, the swallowing latency is prolonged, the timing of swallow in the respiratory cycle is delayed, and consequently, the SW-I pattern is increased.

## Author contributions

Substantial contributions to the conception or design of the work: YO, KD, and RT. Substantial contributions to the acquisition of data for the work: NY, SN, YY, JK, and AI. Substantial contributions to the analysis, or interpretation of data for the work: NY, YO, and SN. Drafting the work or revising it critically for important intellectual content: NY and YO. Final approval of the version to be published: NY, YO, SN, YY, JK, AI, KD, and RT. Agreement to be accountable for all aspects of the work in ensuring that questions related to the accuracy or integrity of any part of the work are appropriately investigated and resolved: NY, YO, SN, YY, JK, AI, KD, and RT.

### Conflict of interest statement

The authors declare that this study received funding from CareIdo Co., Ltd. and J Craft Co., Ltd. The funders were not involved in the study design or collection, analysis, or interpretation of the data. This study was conducted under industry-academia collaboration contracts among Hyogo College of Medicine, Kyoto University, Kobe University, Hiroshima Prefectural University, Foodcare Co., Ltd., CareIdo Co., Ltd., J Craft Co., Ltd., Murata Manufacturing Co., Ltd., and EuSense Medical Co., Ltd.
